# Good ideas for teaching: Design and implementation of the communication workshop “me as team member” for third-year medical students

**DOI:** 10.3205/zma001680

**Published:** 2024-06-17

**Authors:** Annemarie Minow, Katharina Gandras, Josefin Wagner, Jürgen Westermann

**Affiliations:** 1 Universität zu Lübeck, Referat Studium und Lehre Humanmedizin, Lübeck, Germany; 2 Universität zu Lübeck, Institut für Anatomie, Lübeck, Germany

**Keywords:** medical education, curriculum development, interprofessional education, interdisciplinary communication, communication methods

## Abstract

**What is the context informing the project?:**

Effective communication within a medical team is crucial not only because it results in higher job satisfaction and better joint decision-making among team members, but also because, ultimately, it makes for high-quality, patient-centered care. Since the transition to the clinical phase of study poses a challenge for many medical students, the University of Lübeck introduced “Ich im Team” *(me as team member)*, a German-language communication workshop for third-year medical students, in the 2020/21 winter semester.

**Why was the project started?:**

The workshop forms a basis for future collaboration and is meant to strengthen the interpersonal skills needed for working in teams, communicating with patients, and supporting a no-blame culture.

**How is the project carried out?:**

This workshop, which incorporates elements of improvisational theater and coaching, was offered for the first time in 2020/21. Due to the positive evaluations, it has been a required component of the curriculum since the 2021/22 winter semester.

**How is the project evaluated?:**

The students have accepted the workshop very well, which is reflected in the excellent evaluations of it. Furthermore, a research study carried out during the first two times the workshop was conducted showed, among other things, directly positive effects on the ability to work in interprofessional teams and handle mistakes.

**Final overall assessment and outlook:**

The workshop offers students a solid point of entry into the clinical setting and an awareness of their own role on a given team. Covering the content in more depth and the possible inclusion of other study programs are being discussed.

## Zusammenfassung

**Was ist der Kontext bzw. Hintergrund des Projekts?:**

Eine effektive Kommunikation in Behandler-Teams ist von entscheidender Bedeutung, da sie nicht nur zu einer höheren Arbeitszufriedenheit der Teammitglieder und einer besseren gemeinsamen Entscheidungsfindung, sondern letztendlich zu einer qualitativ hochwertigen und patientenzentrierten Versorgung führt. Da der Übergang in den klinischen Studienabschnitt für viele Medizinstudierende eine Herausforderung darstellt, führte die Universität zu Lübeck im Wintersemester 2020/2021 einen Kommunikationsworkshop „Ich im Team“ für die Medizinstudierenden im 3. Studienjahr ein.

**Warum wurde das Projekt begonnen?:**

Der Workshop schafft durch die Auseinandersetzung mit der eigenen Rolle im Team eine Basis für die spätere Zusammenarbeit und soll zwischenmenschliche Kompetenzen wie Teamfähigkeit, Patientenkommunikation und Fehlermanagement stärken.

**Wie wird das Projekt durchgeführt?:**

Der Workshop mit Elementen des Improvisationstheaters und Coachings wurde 2020/21 erstmalig angeboten. Aufgrund der positiven Evaluationsergebnisse ist er seit dem Wintersemester 2021/2022 verpflichtender Bestandteil des Curriculums.

**Wie wird das Projekt evaluiert?:**

Die Studierenden haben den Workshop sehr gut angenommen, was sich in den exzellenten Evaluationsergebnissen widerspiegelt. Eine wissenschaftliche Begleitstudie während der ersten beiden Durchgänge zeigte zudem unmittelbare positive Effekte u.a. auf die interprofessionellen Teamfähigkeiten und den Umgang mit Fehlern.

**Abschließende Gesamteinschätzung und Ausblick:**

Die Workshops bieten für die Studierenden einen guten Einstieg in die Klinik und eine Sensibilisierung für ihre eigene Rolle in Teams. Eine inhaltliche Vertiefung und die Möglichkeit weitere Studiengänge zu beteiligen, werden diskutiert.

## 1. What is the context informing the project?

Effective communication between medical professionals is crucial to prevent medical errors and increase patient safety [1]. Clear and respectful communication also increases job satisfaction in healthcare. Studies show that young physicians and nursing staff in the hospital setting are already affected by burn-out symptoms. However, positive interprofessional cooperation acts as a protective factor in regard to the satisfaction and health of hospital workers [2], [3]. For more than 10 years, the WHO has promoted the integration of interprofessional topics into medical curricula in order to strengthen healthcare systems worldwide [4]. The project described here focuses on third-year medical students because the interactive elements in its teaching strategy encourage active engagement with the content through immediate interaction with others compared to the passive classroom setting [5].

## 2. Why was the project started?

The University of Lübeck’s medical school aims to train students to be physicians who do not just earn good grades, but who also have excellent social and communication skills so that they are able to meet the growing demands of the many inter- and intra-psychic aspects of their profession [6].

The transition from the preclinical to the clinical study phase pushes these interpersonal and intrapsychic skills – teamwork, patient communication, error management, handling stress and so on – increasingly into the foreground. For many medical students this represents a major challenge since basic scientific and mostly theoretical knowledge is taught during the first four semesters of medical study.

In the 2020/21 winter semester, the medical school's head of studies introduced “me as team member”, a communication workshop incorporating elements of improvisational theater and coaching, to prompt an exploration of students’ own roles in mono- and inter-professional teams. This workshop is part of the efforts to improve the teaching of communication skills. During the workshop, students are encouraged to reinforce their interpersonal and communication skills in a practical setting. The workshop is meant to lay a foundation for future cooperation on interprofessional teams, in that the participants intensively grapple with their own separate roles on a team and develop an initial understanding of the importance of interprofessional teams. Moreover, practicing effective communication is supposed to better prepare student for the challenges of the clinical study phase. The selected methods of improvisational theater, stemming from theater pedagogy [7], are already widely applied in the (school) teaching setting [8] and can also be applied in higher education to foster effective communication.

## 3. How is the project carried out?

The communication workshop was offered for the first time in the 2020/21 winter semester on a voluntary basis for third-year medical students and is led each year by the same two external actors and communication coaches, paid for by funds from the medical school's head of studies. It takes place off-campus on one full day (9:00 a.m. to 5:30 p.m., with breaks). The workshop schedule, the learning objectives and a description of the methods are presented in attachment 1 .

During the 2020/21 winter semester, 56 students attended the one-day workshop, broken down into small groups of 12 to 16 participants. Due to the extremely positive student evaluations and the findings from the simultaneously conducted research study, the workshop became a permanent curricular component and a required course for all third-year medical students as of the 2021/22 winter semester. In the winter semesters of 2021/22 (205 attendees) and 2022/23 (210 attendees), the workshop was conducted for small groups of 10-12 participants. Two small groups can be accommodated on one day.

The workshop offers a wide variety of content which is not only taught in a small-group setting, but also specifically tried out and actively explored by the students. Using interactive elements, the content becomes something that can be experienced directly and immediately, and the participants have the opportunity to practice relevant skills in a safe environment. Belonging to the topics covered in the workshop are the ability to work on a team, communication (with a focus on the targeted use of communication strategies), reflecting on roles, coping with stress and handling mistakes. The workshop also attempts to reinforce participants’ self-confidence.

The following questions are focused on in the workshop:

- How do I find my bearings on a pre-existing team?
- How can I grow into my new role?
- How do I cope with stressful situations? How do I look after myself?
- What happens when I make a mistake?
- How can I act with more self-confidence and communicate more effectively?

On the day of the workshop, warm-up games and exercises are used to get acquainted and create an open, trusting group atmosphere. Handling mistakes is addressed and practiced using specific activities. Other exercises focus on being present, self-perception and the perception of others. Methods from improvisational theater and coaching play a central role to promote spontaneity and reactions to one another. In addition, the participants are shown how they can give constructive feedback. As a way to make the learning experience a sustainable one, the participants are given a takeaway sheet summarizing the workshop’s main points and receive three other pieces of writing afterwards, at two-month intervals, with tips and prompts on how to integrate and deepen the workshop’s topics and exercises in their daily lives.

## 4. How is the project evaluated?

This teaching project was accompanied from 2020 to 2022 by a research study. As of the 2022/23 winter semester, student evaluations of the workshop have been administered by the central office for course evaluations.

The study used a pre-post design to survey participants about the ongoing development of their social and personal skills in interprofessional cooperation and handling stress. The study took place in 2020/21 and recorded data at three time points (before the workshop, directly after the workshop, 8 months after the workshop). The questionnaires used included, among others, the Interprofessional Socialization and Valuing Scale (ISVS) [9] and the Error Orientation Questionnaire (EOQ) [10].

In both of the workshops held in 2020/21 and 2021/22, it was possible to measure positive direct effects of the workshop on the ability to deal with spontaneously occurring errors, a subscale of the EOQ, and the ability to work on an interprofessional team, captured by the ISVS. A value of p<0.01 was valid for the findings from both workshop rounds. However, these effects could not be statistically confirmed 8 months later. It is possible that transferring the acquired skills to a non-academic setting, to future clinical practice continues to remain challenging. Moreover, we conclude that a stronger and repetitive embedding of the workshop’s content into clinical teaching is necessary for sustained retention of the acquired skills.

**Figure 1:**
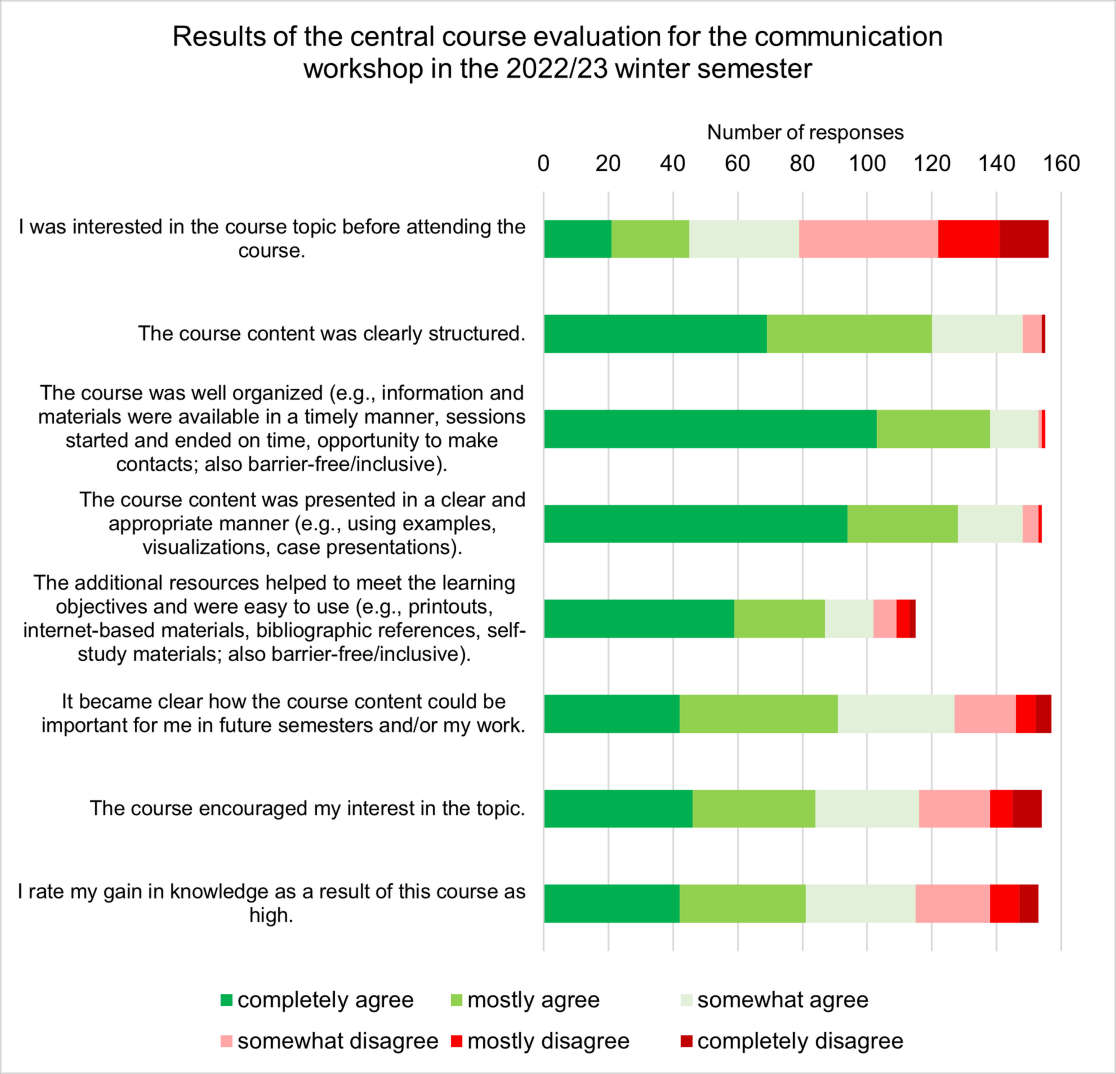
Evaluation results for the communication workshop “me as team member” (2022/23 winter semester)

Positive evaluations of the workshop were also communicated to us by the central office for course evaluations (2021/22: response rate 54%, overall rating 2.1; 2022/23: response rate 77%, overall rating 1.9). The quantitative results for the 2022/23 winter semester are presented in figure 1 (Fig. 1).

Each year since the first workshop was held there has been and continues to be minor adaptation of its content. In the beginning some of the content needed to be shortened (e.g., status within the team according to Keith Johnstone). The subtracted content has been offered since the 2022 summer semester in an optional in-depth seminar called “Ich bin Chef und wer bist du?” *(I’m the boss and who’re you?)*, which is aimed at all of the medical section study programs (medicine, nursing, physiotherapy, midwifery, occupational therapy, speech therapy), as well as psychology students.

Many who participated in the communication workshop wrote in the free-text part of the course evaluation that they wanted similar courses more often and also with students in other healthcare professions. For this reason, the expansion of the project is being considered along with the inclusion of other study programs in the university’s medical section, whereby the requirements regarding content and scheduling for each study program would also need to be taken into consideration.

In addition to the written evaluations at the end of the workshop, there is also a detailed feedback round between the instructors and students about whether the topics had been sufficiently covered and if the workshop’s objectives were achieved.

## 5. Final overall assessment and outlook

The introduction and implementation of the communication workshop was and is a successful project. It offers added value to students because they not only acquire additional medical expertise, but also an awareness for teamwork while gaining self-confidence and familiarizing themselves with the significance of the many roles in the medical profession. Last but not least, the evaluations show that being off-campus and away from the lecture hall, in particular, made the workshop special and as such has the potential to remain in the students’ memories. Given this context, the organizational efforts to plan the course and the costs, both financially and in terms of human resources, to implement this project are worthwhile. The positive experiences with the teaching project and its evaluation results suggest that similar offerings, for and with other study programs, could be of great benefit.

## Authors’ ORCIDs

- Annemarie Minow: [0000-0002-7836-1127]
- Jürgen Westermann: [0000-0001-9054-8755]

## Acknowledgements

During the 2020/21 winter semester this project received funding from the Ärzteverein zu Lübeck e.V.

## Competing interests

The authors declare that they have no competing interests. 

## Supplementary Material

Workshop Schedule for “me as team member”
