# Supplementary material for: Good ideas for teaching: Design and implementation of the communication workshop “me as team member” for third-year medical students
Source: GMS J Med Educ. 2024 Jun 17;41(3):Doc25. doi: 10.3205/zma001680 (PMC11310785; doi:10.3205/zma001680)
Supplement: Workshop Schedule for “me as team member” [file JME-41-25-s-001.pdf]

Attachment 1: Workshop Schedule for “me as team member”

Time	Topic	Learning objective	Description of methods & theatrics	Small groups/Both groups together
**9:00 – 9:30**	Welcome & introduction	Overview of the day’s schedule, agreements for the day are reached together	Brief introduction of the instructors; explanation of the workshop’s concept; joint negotiation of agreements that will be valid for the day; questions from students about the topics in terms of their (future) work in a hospital/clinical setting (these topics will form a reference point over the course of the seminar); statement of intent for the day (internal positive attitudes of the participants toward the topic).	Both groups together
**9:30 – 10:30**	Warm up activities & check-in	Participants meet each other, create awareness and group identity, build trust, deal with stress and mistakes through play	**Coaching tools:**<br>**Getting acquainted via speed dating** – small talk, but different – how to use questions about life or compliments to create a basis of trust for a conversation<br>**Getting acquainted via postcards or an object** – the possibilities at the personal and emotional level of communication, staying curious about the stories of my interlocutor. How do I break the ice before a potentially difficult conversation?<br>**Tools of improvisational theater:**<br>**Circle of energy: “hey!” –** Training how to receive/send<br>**Mindfulness game: “Blinking”** – switching places by blinking – communication begins with the eyes<br>**Switching places in the circle by name** – how do I deal with topics like sending/receiving and stress?<br>**Circle game: “Yeehaw”** – Attentiveness and using energy for the group.	Both groups together

**10:30 – 11:00**	Exercises to lay the basis for being present	Activate presence, perception of self and others, non-verbal communication<br>“one cannot not communicate”<br>(P. Watzlawik)	**Tool from the practice of being present:**<br>Starting point: “neutral” position<br>Feel the ground under your feet, notice the space around you.<br>Take time, breathe – very consciously, especially in stressful situations, create a mindfulness of your own, usually unconscious, body language.<br>**Presence training:**<br>Turn on imaginary headlights and shine them out into the room.<br>Establish contact with the other person, even before anyone speaks.<br>Our bodies ALWAYS speak too.<br>Our eyes (eye-contact) say more than a thousand words.<br>Send language, especially when wearing a medical mask.<br>Take responsibility for having what you say actually reach the recipient.	Both groups together
**11:00 – 12:00**	Team role play	Capacity for teamwork, communication and communication strategies (moderating, delegating, focusing, reflecting on roles, etc.)	**Coaching tool: Team dynamics combined with improvisational acting:**<br>Construct a tableau based on a slogan – a team organizes itself in 3 minutes (from there on with defined team roles).<br>Discussion of and reflection on what was experienced and the scope of action available to the different team roles.	Both groups together
**12:00 – 1:30**	“I” exercise	Practice being present, perceiving one’s self, perceiving others, what does my appearance say about me? My posture? My facial expression?<br>Giving and receiving constructive feedback.	**Coaching tool from the practice of being present, derived from the systemic coaching tool “forum”:**<br>Perceiving others vs. perceiving one’s self<br>**Focus A on the actively sending person**<br>**Focus B on observing and describing the audience/recipients**<br>(based on the four-sides model by F. von Thun and the constructivist coaching approach by P. Watzlawik: It’s not what you “are”, it’s what you “do”).	Small groups

			**Description of the exercise:**<br>The performer acts out a scene according to simple and clear rules.<br>This is about presenting yourself, to show and consciously notice the performative situation. How is it for me to be the focus? What does that do to me? How does my body react?<br>The recipients practice reflecting back: observe, perceive and then describe.<br>Clarification of terms: description vs. interpretation.<br>Afterwards, individualized advice is given for a relaxed and more self-confident performance. All of the participants take the role of performer once and the rest of the time are the audience/recipients.	
**1:30 – 2:30**	Break			
**2:30 – 3:30**	Improvisation training	Trying out improvisational techniques, creativity, spontaneity, reacting to one another, improvising common scenes in a hospital and life, dealing playfully with stress and clichéd roles, attentively reading the situation in the room and the people around me.<br>Reflecting on the scenarios and discussing the different latitudes and room for maneuver, especially in systems with strong hierarchies, shifting the focus to self-empowerment in conflict situations.	**<u>Tools of improvisational theater (Impros and Mirroring)</u>**<br>**<u>Warm-ups:</u>**<br>Circle activity: “I am a tree”:<br>Freeze-frames are spontaneously created by 3 people, with rapid changes,<br>Example:<br>Actor 1: I am a nervous patient.<br>Actor 2: I am an angry syringe.<br>Actor 3: I am a flustered medical resident.<br>Let the situation take shape and have its effect.<br>Actor 1 takes actor 2 or 3 with them.<br>A new 3-person group starts with the actor who remains.<br><br>Circle exercise:<br>"Rumpelstiltskin" - Sentence is spoken and performed word for word around the circle in different ways.	Small groups

			Noticing and practicing dramatic means of expression, such as the posture and stance, mindset and physical state of my character, emotions and their physical expression, the acting process (actions of the character).<br>Improvising scenes as is done in improvisational theater (setting, relationship, conflict, resolution):<br>Participants share challenging situations in the hospital setting. Scenes are improvised by 2-4 people using the tools above. Audience describes what is seen, active discussion about it, solutions are looked for, suggested and tested by acting them out.	
**3:30 – 4:30**	Final exercise: “emotional waiting room”	Training attentiveness, raising awareness of the person in front of me, offering options, mimicking, escalating, leaving your comfort zone, creating situations together.	**Tool used in improvisational theater:**<br>**“emotional elevator”:** Four chairs, on each of which an actor sits. There is a predominant emotion/state that amplifies in expression from chair 1 to 4. (Example: somewhat nervous, nervous, very nervous, panicked.)<br>Improvisation is added to this with the instruction that when actor 4 leaves the room, everyone moves over one chair and a new actor on chair 1 joins them. Start again with a new emotion, new attitude, new scene.	Both groups together
**4:30 – 5:30**	Closure & feedback	Which topics are still unfinished? Reflecting back the learning objectives, feedback round.	**Coaching tool: team dynamics:**<br>Final circle, check-out<br>3 prompts for feedback, symbolized by 3 objects:<br>Rock – stands for challenging moments,<br>Key – stands for a key or light-bulb moment,<br>Heart – stands for favorite moments.<br><br>Closing words of the seminar leader, reflection of the group dynamic, further prompts.	Both groups together

Note: A 10-minute break is taken in the morning and afternoon, as needed.
